# Organizational cynicism and job burnout: a moderated mediation model of work alienation and leader-member exchange

**DOI:** 10.3389/fpsyg.2026.1910570

**Published:** 2026-09-28

**Authors:** Xiuqin Zhou, Zhong Han, Zengjun Sun

**Affiliations:** 1 School of Labor Relations, Shandong Management University, Jinan, Shandong, China; 2 School of Education, Weifang University of Science and Technology, Weifang, Shandong, China; 3 School of International Circulation, Namseoul University, Cheonan, Chungcheongnam-do, Republic of Korea

**Keywords:** employee, job burnout, leader-member exchange, organizational cynicism, work alienation

## Abstract

**Background:**

In organizational management practice, understanding the intrinsic link between employees’ negative perceptions of the organization and their state of emotional exhaustion, as well as identifying the transmission mechanisms and boundary conditions, has become a key issue in mitigating the risk of job burnout. However, the mechanisms through which organizational cynicism relates to work alienation and job burnout, in addition to the moderating role played by the leader-member exchange in this relationship, still lacks clear theoretical explanations and empirical tests.

**Method:**

Based on Conservation of Resources theory, this study developed a moderated mediation model to systematically examine the relationships among the variables. Using convenience sampling in cross-sectional survey, this study collected valid questionnaire data from 658 employees across 27 enterprises (varying in ownership type and scale) and conducted regression analysis and moderating effect analysis using SPSS.

**Results:**

According to the results, organizational cynicism and job burnout were significantly positively correlated, and the indirect effect of organizational cynicism on job burnout via work alienation was statistically significant. The results of the moderation analysis indicated that leader-member exchange moderated the relationship between organizational cynicism and work alienation, as well as the relationship between organizational cynicism and job burnout, in a negative manner. In addition, employees’ job burnout differed across groups defined by gender, age, educational background, years of working, and weekly working hours.

**Conclusion:**

The research results provide theoretical basis and practical inspiration for organizations to identify high-risk groups and design stratified intervention strategies to alleviate the risk of job burnout.

## 1 Introduction

Against the background of deep digital and intelligent penetration into organizational environments, accelerated work pace, intensified market competition, and continuously rising information processing loads are profoundly reshaping the modern occupational ecosystem. These changes collectively translate into a source of stress that cannot be ignored in the field of employee mental health (Zeng and Hu, 2024). Job burnout (JB) refers to a psychological syndrome formed by individuals under long-term work pressure, marked by depersonalization, feelings of emotional exhaustion, and a lower sense of personal achievement (Maslach et al., 2001). This state is usually accompanied by employees’ impaired physical and mental well-being together with reduced work engagement (Chen et al., 2026; Zhang et al., 2024). An empirical study focusing on 8,018 employees in the telecommunications industry in Shandong Province, China, showed that over 50% of respondents exhibited varying degrees of JB (Song et al., 2025). This high prevalence highlights the urgency of systematically identifying and intervening in JB. It is worth noting that with the continuous intensification of technological iterations, process reengineering and performance pressure in the intelligent era, employees are more likely to perceive the gap between organizational promises and reality, and form systematic doubts about the organization. This kind of negative cognitive rigidity may be more resource-draining than work-related stress alone and is more deeply linked to JB. Therefore, systematically identifying and understanding the composition and manifestations of JB is not only a fundamental prerequisite for improving employee mental health and work experience but also a practical requirement for organizations to maintain sustainable competitiveness and achieve human-centered management goals in the intelligent era.

The severity of JB has become increasingly prominent and has drawn widespread concern from the academic community. However, existing research has mostly focused on single dimensions such as negative interpersonal behaviors (Ullah and Ribeiro, 2024), job characteristics (Lei et al., 2025; Xie et al., 2024), or organizational support (Al’Ararah et al., 2024; Dara et al., 2025), with fewer studies systematically integrating the joint actions of organizational cognition, individual psychological processes, and leadership contextual factors within the same theoretical framework. To this end, this study constructed an integrated analytical framework to systematically examine the relationship mechanisms among organizational cynicism, work alienation, leader-member exchange (LMX) and employees’ JB. It is also worth clarifying at the outset that our empirical context is China, a cultural setting where leader–member dynamics are often permeated by collectivist values, Confucian role expectations, and guanxi-based relational norms. While the core mechanisms of Conservation of Resources (COR) theory are presumed to be universal, the strength and functional form of relational moderators such as LMX may be culturally contingent. We therefore acknowledge this cultural boundary upfront and revisit its implications in the discussion, rather than treating our findings as culturally unqualified.

According to the core tenet of the COR theory (Hobfoll, 1989), individuals always strive to acquire, maintain, and protect their valuable resources. When actual resource loss occurs, or when individuals fail to obtain expected returns after investing resources, these situations are closely associated with psychological stress and burnout responses. Under this theoretical framework, there are close correlations among organizational cynicism, work alienation, LMX and JB. Specifically, organizational cynicism reflects employees’ deep disappointment and skepticism regarding the organization’s fairness, integrity, and sincerity toward change. This negative perception of the organization in itself constitutes a significant drain on psychological resources. When employees remain in a state of critical scrutiny of organizational motives and decisions, they exhibit high levels of organizational cynicism and correspondingly higher levels of JB (Kim et al., 2025). Meanwhile, when employees suffer from resource losses, they tend to activate resource protection strategies by psychologically distancing themselves from their work roles. A stronger sense of work alienation is associated with greater difficulty in deriving resources from work (e.g., sense of accomplishment, meaning, social connection), and with a more pronounced net loss of those resources. Under this condition, the work alienation tendency shown by employees demonstrates a significant and consistent correspondence with their level of JB (Galal et al., 2026). More critically, LMX, as a key social context resource, reflects the richness of emotional support, trust, autonomy and information resources provided by superiors to employees. With the infusion of these external resources, employees’ depleted psychological resources tend to be replenished, and their JB may consequently decrease (Wallace and Coughlan, 2023).

Although prior literature has separately examined the pairwise relationships among organizational cynicism, work alienation, LMX, and JB, these studies have largely treated these constructs as discrete phenomena rather than as interconnected nodes within a resource-based motivational system. Moving beyond the mere introduction of new constructs, this study fundamentally rethinks the conditional architecture that governs these established interrelationships. Specifically, we propose an integrative moderated mediation framework that systematically links these constructs into a unified process model. Additionally, unlike previous studies that predominantly focused on direct attitudinal associations between cynicism and burnout, we conceptualize work alienation as the key psychological transformation mechanism that accounts for a substantial portion of the relationship between organizational cynicism and individual resource depletion. Meanwhile, we reposition LMX from a general protective factor to a contextual resource-switching moderator that is empirically associated with whether cynical employees show a greater tendency toward resource conservation or reinvestment. Critically, our analytical strategy extends beyond conventional subgroup estimations; we employ the Johnson-Neyman technique to identify the precise boundary conditions under which LMX is operative, thereby offering a more granular delineation of when the conditional process holds than is available in prior pairwise research. By integrating these dynamics within the COR framework, this study moves beyond simple variable-combination to offer a nuanced explanatory mechanism for why some cynical employees burn out while others do not, thereby providing a more rigorous and practice-oriented basis for mitigating employee burnout in contemporary organizations.

### 1.1 Theoretical basis

Drawing upon the COR theory (Hobfoll, 1989), this study proposes an integrated framework linking organizational cynicism to JB through work alienation. COR theory posits that individuals are fundamentally motivated to protect their existing resources and invest in new ones to prevent future loss. From this perspective, organizational cynicism is conceptualized not merely as a static negative attitude, but as a defensive resource investment strategy. When employees perceive that their invested resources (e.g., trust, loyalty, and emotional commitment) fail to yield expected returns in an unjust or volatile organizational environment, they strategically withdraw these resources to prevent further depletion. This cynical disengagement severs employees’ psychological connection to the organizational resource caravan, thereby blocking their access to associated contextual resources, such as informational support, recognition, and collaborative opportunities. As a result, this disruption prevents the initiation of a potential gain spiral while initiating a loss spiral in which resource depletion accumulates over time (Hobfoll, 2001). In a state of continuous resource depletion, individuals exhibit organizational cynicism and a higher level of JB, which reflects a defensive adaptive tendency to preserve remaining resources. Work alienation involves the obstruction of the resource investment process, wherein employees reduce their investment of meaning and effort in work, and fail to generate the resource returns typically associated with task engagement. This condition coexists with the deepening of the loss spiral and the restricted initiation of a gain spiral, with symptoms of burnout gradually emerging in this process. LMX as a social exchange resource within organizations, is associated with employees’ access to organizational resources, emotional support, and trust returns. It constitutes a protective context for the resource caravan and a critical resource passageway, and is linked to the resource depletion state and the progression of the loss spiral triggered by organizational cynicism. Meanwhile, emotional and informational resources derived from leaders can compensate for existing losses and are associated with the creation of new resource gains.

### 1.2 Organizational cynicism and job burnout

Organizational cynicism refers to employees’ persistent negative attitudes toward their organization and its management practices, often manifested as disparaging evaluations of organizational decisions, distrust of organizational motives, and emotional detachment and contempt (Dean et al., 1998). Previous studies have shown that organizational cynicism is significantly positively associated with JB (Kim et al., 2025). For instance, the study by Naseer et al. (2021) confirmed that there is a positive and significant association between organizational cynicism and emotional exhaustion. The emotional detachment and loss of meaning associated with organizational cynicism are often accompanied by lower employee engagement and a tendency toward negative and avoidant work behaviors. Under such conditions, employees have fewer opportunities to acquire new resources from work, and the characteristics of JB emerge (Salama et al., 2025). Furthermore, in a highly cynical organizational environment, employees will question the true motives of organizational policies, show a reduced willingness to take initiative, and limit their own possibility of obtaining and accumulating work resources. For this reason, organizational cynicism is considered a key cognitive risk factor in the onset and persistence of JB (Karaman et al., 2025). In sum, the resource-depleting nature of organizational cynicism establishes the foundation for the transformation of engaged employees into burnt-out individuals.

### 1.3 The mediating role of work alienation

Work alienation refers to a state in which an individual experiences psychological separation and detachment from their work role or work environment, manifested as a lack of meaning in work, a sense of distance and passivity toward work activities, and a disconnection between the self and work activities (Kanungo, 1982). Recent related research has found that organizational cynicism is significantly positively associated with work alienation (Demir and Inan, 2025). When employees continuously question the authenticity of organizational policies and belittle management practices, their psychological connection with the work itself gradually breaks, and the work is merely regarded as a passive task under external demands rather than a meaningful activity with intrinsic value. In this situation, employees not only lack the motivation to engage proactively, but also find it difficult to derive emotional fulfillment and replenish their energy from their work, maintaining a persistently high level of work alienation (Singh and Randhawa, 2022). For example, Jiang et al. (2019) discovered that frontline production employees with high organizational cynicism tend to disparage the true motives behind company management decisions and hold skeptical attitudes toward organizational policies, thereby maintaining psychological distance from their work roles and exhibiting higher levels of work alienation. It can be seen from this that employees with organizational cynicism in the organization are psychologically drifting away from their job roles, and work alienation has become their typical psychological state at work.

Furthermore, existing research has indicated a significant positive association between work alienation and JB (Galal et al., 2026). Specifically, employees with a strong sense of work alienation tend to approach their job responsibilities with a cold and evasive attitude. Under such a negative coping strategy, a decline in employees’ commitment to their work tasks, along with reduced opportunities to draw psychological and emotional resources from colleagues’ support and task completion, often accompanies the onset and progression of burnout (Ferreira and Gomes, 2023). The lack of meaning and psychological detachment brought about by work alienation make it difficult for employees to establish stable emotional connections and sustained resource circulation mechanisms within the work environment, with this breakdown in emotion and resources manifesting as emotional exhaustion (Guo et al., 2022). Meanwhile, prior research has also confirmed that there is a positive correlation between organizational cynicism and teachers’ JB through work alienation (Demir and Inan, 2025). Therefore, an in-depth examination of the mediating role of work alienation between organizational cynicism and employees’ JB can help theoretically elucidate how negative perceptions of the organization co-occur with psychological detachment from work in the development of burnout, and provide empirical guidance for organizations seeking to alleviate employee alienation and prevent JB.

### 1.4 The moderating role of leader-member exchange

LMX refers to the dyadic interactional relationship of varying quality that develops between a leader and a subordinate through a role-making process (Dansereau et al., 1975). Existing studies have pointed out that there is a significant negative correlation between LMX and organizational cynicism, work alienation, and JB (Turkmenoglu et al., 2022; Wallace and Coughlan, 2023; Wu et al., 2025). Although organizational cynicism and employee work alienation are typically highly correlated, this relationship may vary depending on the quality of LMX. In contexts of authoritarian leadership, for example, organizational cynicism among employees correlates more strongly with work alienation (Jiang et al., 2019). Existing research also confirms that LMX has a buffering effect between stressors and JB. For example, Li et al. (2024) found that in the contexts of high-quality LMX, employees exhibit higher levels of emotional and resource support, while the positive relationship between emotional labor and JB is weaker, and the cumulative path of emotional exhaustion is also lower. This provides theoretical support for the present study’s examination of the moderating role of LMX in the relationship between organizational cynicism and JB. Although existing research has initially explored the buffering mechanism of LMX on employees’ negative attitudes and behaviors, systematic studies on the specific moderating paths between organizational cynicism and work alienation, as well as JB, are still relatively limited. Therefore, this study aims to focus on LMX, a key organizational contextual resource, to further elucidate the mediating mechanisms in the process associated with organizational cynicism, with the goal of providing a more targeted theoretical foundation for organizational behavior interventions and human resource management practices.

### 1.5 This study

Drawing upon the COR-based framework established in Section 1.1, this study investigates the relationship between organizational cynicism and JB, with a particular focus on the mediating role of work alienation and the moderating role of LMX. By integrating these variables, we aim to theoretically delineate the joint mechanism of organizational negative cognition, psychological resource loss processes, and leadership support in the formation of JB, thereby offering a comprehensive model for both academic understanding and practical intervention. Based on this, the study proposes the theoretical model (Figure 1) and makes the following assumptions:

**Figure 1:**
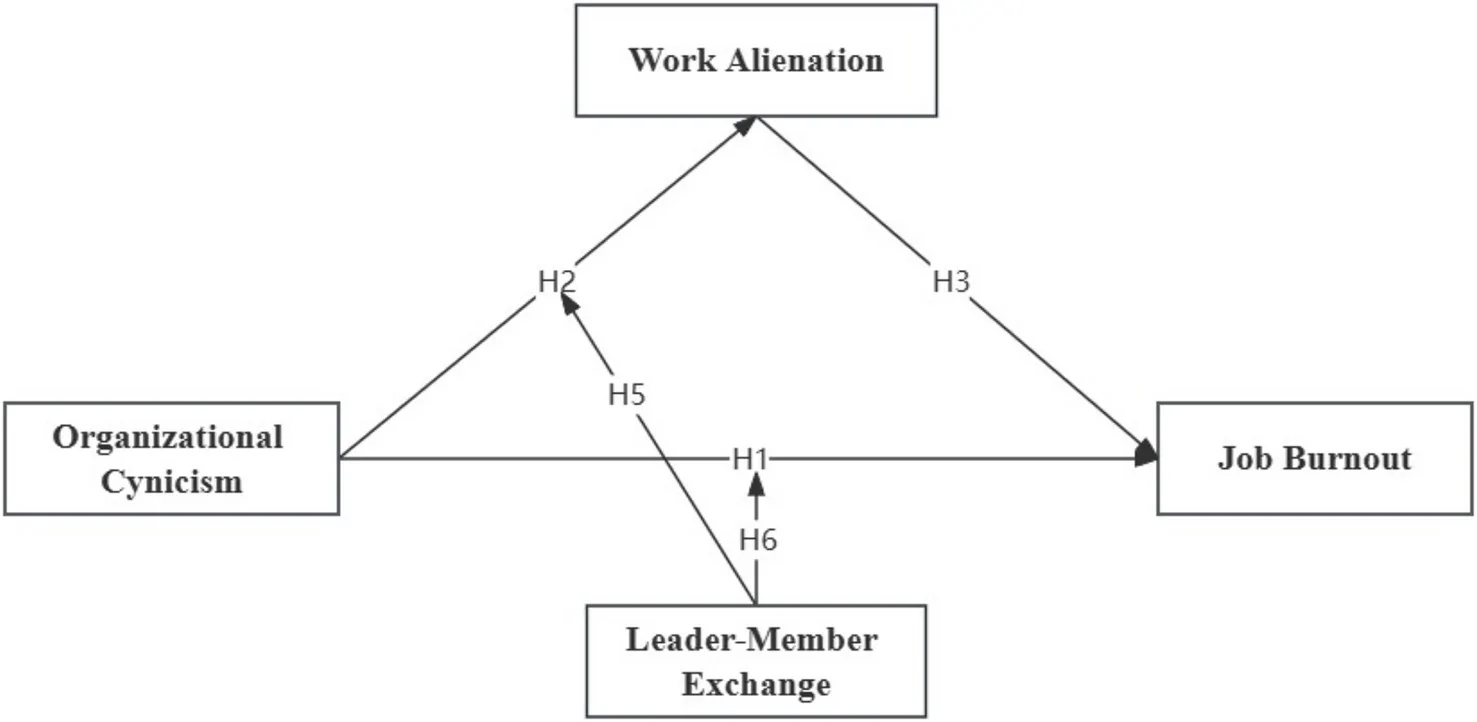
Theoretical model. H4 denotes the indirect effect of organizational cynicism on JB via work alienation.

*H1*: Organizational cynicism is significantly positively associated with JB.

*H2*: Organizational cynicism is significantly positively associated with work alienation.

*H3*: Work alienation is significantly positively associated with JB.

*H4*: Work alienation mediates the relationship between organizational cynicism and JB.

*H5*: LMX moderates the relationship between organizational cynicism and work alienation.

*H6*: LMX moderates the relationship between organizational cynicism and JB.

## 2 Methods

### 2.1 Data collection and sample

This study conducted online data collection through a digital survey platform^1^ from May to June 2026. Considering that it is difficult to obtain a complete national list of employed employees and that the main purpose of this study is to test the theoretical relationship between variables rather than to estimate the overall parameters, a convenience sampling strategy was adopted. At the same time, the sample exhibits some heterogeneity across key demographic variables, the use of convenience sampling in this study is acceptable to a certain extent (Landers and Behrend, 2015). It should be noted that because the sample is not a strictly probabilistic sample, caution should be exercised when generalizing the findings to the population. The specific implementation process is as follows: The research team, relying on the local industrial park management committee, multiple human resource service agencies, and the researcher’s social network, proactively contacted enterprises from different economic regions within the country. In selecting these enterprises, the team prioritized accessibility through these channels and the organizations’ willingness to participate, rather than employing a random sampling procedure. At the organizational level, a total of 27 enterprises agreed to participate, covering various ownership types such as state-owned, private, and foreign-funded, as well as different scales (small, medium, and large). After obtaining verbal or written permission from the heads of human resources departments at each company, the research team distributed survey links to eligible current employees (excluding those employed solely through labor dispatch agencies) via easily accessible channels, including targeted forwarding by internal HR departments, invitations to work groups, scanning QR codes to complete the survey in campus cafeterias or break rooms. The survey invitation was sent to or made accessible to approximately 700 employees. Ultimately, 674 employees accessed the questionnaire link and completed the survey. All participants were informed in detail about the research purpose, content, privacy protection measures, and the voluntary principle. Only after clicking to read and voluntarily signing the electronic informed consent form could they enter the formal questionnaire filling stage. This research protocol has been approved by the Shandong Management University Ethics Review Committee (Approval No.: 26001).

The questionnaire comprised 52 items in total. Following the sample size estimation guideline proposed by Kline (2023), which recommends a minimum of 10 respondents per item, the baseline required sample was 520. Accounting for an anticipated 20% invalidity rate, the target sample size was set at 624. To ensure that the final valid sample would still meet the minimum requirement after the removal of incomplete or careless responses, we over-recruited and ultimately obtained 674 responses. Received questionnaires underwent rigorous screening according to the following sequential exclusion criteria: (1) Missing data screening: Valid responses required at least 80% item completion. Only 2 cases were deleted for exceeding this threshold (i.e., >20% missing). Surveys with incomplete cases falling short of the deletion criterion were treated via multiple imputation. Specifically, this imputation procedure was applied to 6 cases with incomplete responses across 4 key study variables (all demographic variables were complete). (2) Response pattern screening: Questionnaires exhibiting clear extreme response tendencies (e.g., selecting “Strongly Disagree” or “Strongly Agree” for >90% of items) were excluded. 3 cases were removed based on this criterion. (3) Response time screening: Adopting the procedure from Meade and Craig (2012), we established the lower bound at the 3rd percentile (134 s) of all response latencies, excluding cases with times below that point. This criterion resulted in the removal of 11 cases. In total, 16 cases were excluded. This yielded 658 valid cases, representing a usable-response rate of 97.63% based on the 674 returned questionnaires. This final valid sample of 658 comfortably exceeded the minimum requirement and also surpassed the upper bound (558) of the range recommended by Fritz and MacKinnon (2007) for detecting small-to-moderate interaction effects (*β* = 0.14–0.26) with adequate statistical power (≥ 0.80) in moderated mediation models. With 658 valid cases, our sample provides sufficient power for the main, mediating, and moderated mediation paths, as well as for the subsequent Johnson-Neyman boundary identification.

Table 1 provides an overview of the participants’ key characteristics. The distribution of the sample in key variables such as gender (50.15% male and 49.85% female), age (63.98% of young and middle-aged people aged 26–45), and position level (84.35% for both ordinary employees and grassroots managers) is somewhat comparable to the overall characteristics of non-management positions and grassroots positions in the China Labor Statistical Yearbook. It indicated that it had a degree of sample heterogeneity. However, due to the non-probability nature of convenience sampling, this sample should not be regarded as strictly representative of the national employed population; thus, these comparisons are intended only as reference points and should not be over-interpreted as statistical representativeness. Additionally, the educational attainment of this sample (70.21% holding a bachelor’s degree or higher) is higher than the national average, this represents a common bias associated with convenience samples; in subsequent analyses, education background will be included in the model as a control variable to mitigate its potential impact. Given the sample composition, which predominantly consists of frontline and middle-level managers and covers enterprises of diverse ownership types and sizes, the theoretical population to which the findings of this study can be reasonably generalized is primarily confined to frontline and middle-level employees within various types of Chinese organizations, rather than the entire employed population. Within this scope, the heterogeneity of the sample is sufficient to support the examination of relationships among the variables.

**Table 1:** Distribution of sample characteristics.

Demographic and occupational background variables	Category	Quantity	Percentage (%)
Gender	Male	330	50.15
	Female	328	49.85
Age	25 years and below	89	13.53
	26–35 years	211	32.07
	36–45 years	210	31.91
	46–55 years	116	17.63
	56 years and above	32	4.86
Education background	High school/Vocational school and below	67	10.18
	Associate degree	129	19.61
	Bachelor’s degree	300	45.59
	Master’s degree and above	162	24.62
Marital status	Unmarried	113	17.17
	Married without children	173	26.29
	Married with children	328	49.85
	Divorced	36	5.47
	Widowed	8	1.22
Years of working	Less than 1 year	68	10.34
	1–3 years	124	18.84
	4–6 years	118	17.94
	7–9 years	149	22.64
	10 years and above	199	30.24
Department	Production	164	24.93
	Marketing	154	23.40
	Finance	123	18.69
	Research and development	60	9.12
	Human resources	61	9.27
	Administration	46	6.99
	Others	50	7.60
Position level	Ordinary employee	337	51.22
	Front-line manager	218	33.13
	Middle manager	82	12.46
	Senior manager	21	3.19
Weekly working hours (including overtime)	40 h and below	174	26.44
	41–50 h	216	32.83
	51–60 h	185	28.12
	Above 60 h	83	12.61

### 2.2 Measuring instruments

#### 2.2.1 Organizational cynicism

This study used the scale developed by Brandes et al. (1999) to assess employee organizational cynicism. This scale contains 14 items covering three dimensions: affective cynicism, cognitive cynicism, and behavioral cynicism, with an example item such as “I do not see much similarity between what the organization claims to do and what it actually carries out.” Responses were recorded on a 5-point Likert scale, where elevated scores reflected greater intensity of organizational cynicism. This scale has demonstrated good reliability and validity in Chinese samples (Peng et al., 2021). Given the high inter-correlations among the three sub-dimensions (ranging from 0.810 to 0.858) and the theoretical focus of this study on organizational cynicism as a holistic construct rather than its distinct facets, we adopted a second-order factor structure in which the three dimensions loaded onto a single higher-order cynicism factor. The second-order model showed satisfactory fit: *χ*^2^/d*f* = 1.941, RMR = 0.026, IFI = 0.987, RFI = 0.968, GFI = 0.969, CFI = 0.987, NFI = 0.975, TLI = 0.984, RMSEA = 0.038. All first-order factor loadings exceeded 0.7, and the second-order loadings for affective (0.926), cognitive (0.957), and behavioral (0.948) cynicism were all significant, justifying the aggregation of the three dimensions into a composite score.

#### 2.2.2 Work alienation

This study used the scale developed by Nair and Vohra (2009) to assess employee work alienation. This scale contains 8 items, with an example item such as “I often wish I were doing something other than work.” A 5-point Likert scale was used, on which higher point totals signified more pronounced work alienation. This scale has demonstrated good reliability and validity in Chinese samples (Wang and Yuan, 2025). The scale showed satisfactory structural validity (*χ*^2^/d*f* = 1.582, RMR = 0.023, IFI = 0.995, RFI = 0.980, GFI = 0.988, CFI = 0.995, NFI = 0.986, TLI = 0.969, RMSEA = 0.030).

#### 2.2.3 Leader-member exchange

LMX was assessed using the scale developed by Graen and Uhlbien (1995). This scale contains 7 items, with an example item such as “My leader understands my work-related difficulties and needs very well.” A 5-point Likert scale measured LMX, where higher scores meant higher quality. This scale has demonstrated good reliability and validity in Chinese samples (Li et al., 2026). The scale showed satisfactory structural validity (*χ*^2^/d*f* = 2.759, RMR = 0.025, IFI = 0.987, RFI = 0.970, GFI = 0.983, CFI = 0.987, NFI = 0.980, TLI = 0.981, RMSEA = 0.052).

#### 2.2.4 Job burnout

This study used the JB scale developed by Maslach et al. (1997) and revised by Li and Shi (2003). This scale contains 15 items covering three dimensions: cynicism, emotional exhaustion, and professional efficacy. An example item is “I doubt the meaning of the work I do.” A 5-point Likert scale was used, where elevated scores reflected greater severity of JB. Of the 15 items, items 10–15 exclusively measure the professional efficacy dimension and are negatively worded (reverse-keyed). Consequently, we reverse-coded these six items so that higher composite scores uniformly represent more severe JB across all dimensions. This scale has demonstrated good reliability and validity in Chinese samples (Chen et al., 2025). Consistent with the well-established three-factor structure of the Maslach Burnout Inventory (MBI), we specified a second-order factor model with emotional exhaustion, cynicism, and professional efficacy as three first-order factors loading onto a single second-order burnout factor. The second-order model demonstrated satisfactory fit: *χ*^2^/d*f* = 1.822, RMR = 0.025, IFI = 0.987, RFI = 0.965, GFI = 0.970, CFI = 0.987, NFI = 0.971, TLI = 0.984, RMSEA = 0.035. All first-order factor loadings were above 0.7, and the second-order loadings for emotional exhaustion (0.940), cynicism (0.927), and professional efficacy (0.947) were all significant, justifying the aggregation of the three dimensions into a composite score. It should be noted that the professional efficacy dimension was reverse-keyed prior to model estimation; after reverse-coding, all six items loaded positively onto the professional efficacy factor (loadings ranging from 0.719 to 0.786), confirming that the reversed items appropriately captured the intended construct.

### 2.3 Statistical analysis

Data processing in this study was carried out using SPSS 27.0 and AMOS 28.0. Initially, descriptive statistics were computed, after which the reliability and validity of the employed scales were assessed. A confirmatory factor analysis (CFA) was conducted to evaluate measurement model fit and to check for common method bias (CMB). Second, between-group difference analysis was conducted. Finally, hierarchical regression analysis was used to test the mediating effect, and the SPSS PROCESS 4.2 macro (Model 8) was used for moderation analysis. Notably, although CFA was performed within the SEM framework to validate the latent constructs, we intentionally employed the regression-based PROCESS approach for structural path testing rather than full SEM. This analytical choice was made for three specific reasons. First, our hypothesis involves a moderated mediation model with interaction terms; estimating latent-variable interactions in CB-SEM with our sample size often poses a high risk of non-convergence and unstable parameter estimates. Second, PROCESS offers superior algorithmic support for probing conditional effects, particularly the ease of generating simple slopes and conditional indirect effect plots across different moderator levels (Hayes, 2015), which are less accessible in SEM frameworks. Third, using composite scale means in PROCESS substantially reduces model complexity and avoids over-parameterization, allowing us to focus on the robustness of the interaction pattern. We recognize that this approach does not correct for measurement error in structural paths; however, given that our CFA already confirmed satisfactory measurement quality, we consider this trade-off acceptable. This strategy aligns with the analytical distinctions recommended by Anderson and Gerbing (1988), using CFA strictly for measurement quality assurance while leveraging PROCESS for hypothesis testing. Moreover, a 5,000-resample bias-corrected bootstrap was employed to further reduce estimation bias in standard errors, avoiding dependence on rigid normality assumptions and yielding more reliable confidence intervals (Preacher and Hayes, 2008).

## 3 Results

### 3.1 Descriptive statistics and correlation tests

Prior to conducting multivariate procedures, the present work assessed distributional properties through examinations of skewness and kurtosis. Descriptive statistics, namely means (*M*) and standard deviations (SD), were then computed for variables showing normal distributions. According to the criteria of Kim (2013), when absolute skewness is less than 2 and absolute kurtosis is less than 7, the data are considered approximately normally distributed. As shown in Table 2, all variables in this study meet the requirements for a normal distribution.

**Table 2:** Descriptive statistics and correlations.

Variables	*M* ± SD	Skewness	Kurtosis	Organizational cynicism	Work alienation	LMX	JB
Organizational cynicism	2.961 ± 0.844	0.075	−0.806	1			
Work alienation	3.033 ± 0.841	−0.143	−0.866	0.676***	1		
LMX	3.084 ± 0.799	−0.127	−0.878	−0.420***	−0.338***	1	
JB	3.027 ± 0.797	−0.081	−0.860	0.638***	0.666***	−0.301***	1

*N* = 658, LMX, leader-member exchange; JB, job burnout. Path significance: ***p < 0.001, the same below.

The correlation analysis results in Table 2 showed that organizational cynicism was significantly positively associated with work alienation and JB, and significantly negatively associated with LMX. Work alienation was significantly negatively associated with LMX and significantly positively associated with JB. LMX was significantly negatively associated with JB. On the whole, the data supported the theoretically directions of the relationships between the variables.

### 3.2 Testing of validity and reliability

To maintain consistency in factor structure specification across all study variables, each multi-dimensional construct (organizational cynicism and JB) was modeled as a second-order factor, with its respective sub-dimensions as first-order factors. This approach allowed us to respect the theoretical multidimensionality of these constructs, compute composite scores representing the overarching construct in subsequent path analyses, and avoid the statistical and conceptual issues associated with treating multidimensional scales as strictly unidimensional. For unidimensional constructs (work alienation and LMX), standard first-order confirmatory factor models were estimated.

As shown in Table 3, all standardized factor loadings exceeded 0.70 (Hair et al., 2019), and the Cronbach’s alpha coefficients and composite reliability (CR) for all latent variables exceeded the standard threshold of 0.7 (Hair et al., 2021), indicating good internal consistency of the scales. The average variance extracted (AVE) for each latent variable exceeded the recommended criterion of 0.5 (Fornell and Larcker, 1981), confirming good convergent validity of the scales. As shown in Table 4, the heterotrait-monotrait (HTMT) ratios among all latent variables were below the strict threshold of 0.85 (Henseler et al., 2015). Meanwhile, as shown in Table 5, the square roots of the AVE for each latent construct (reported on the diagonal) are larger than their corresponding inter-construct correlation coefficients, satisfying the Fornell-Larcker criterion (Fornell and Larcker, 1981). Taken together, these results confirm that the scale has good discriminant validity.

**Table 3:** Reliability and convergent validity.

Variables	Item	Loading	Cronbach’s alpha	CR	AVE
Organizational cynicism	OC1	0.772	0.943	0.950	0.574
	OC2	0.785			
	OC3	0.747			
	OC4	0.762			
	OC5	0.753			
	OC6	0.755			
	OC7	0.734			
	OC8	0.731			
	OC9	0.758			
	OC10	0.720			
	OC11	0.763			
	OC12	0.738			
	OC13	0.788			
	OC14	0.792			
Work alienation	WA1	0.778	0.889	0.912	0.564
	WA2	0.740			
	WA3	0.713			
	WA4	0.737			
	WA5	0.763			
	WA6	0.782			
	WA7	0.758			
	WA8	0.735			
LMX	LMX1	0.770	0.878	0.904	0.575
	LMX2	0.751			
	LMX3	0.746			
	LMX4	0.776			
	LMX5	0.779			
	LMX6	0.772			
	LMX7	0.710			
JB	JB1	0.727	0.941	0.948	0.549
	JB2	0.731			
	JB3	0.729			
	JB4	0.740			
	JB5	0.723			
	JB6	0.750			
	JB7	0.751			
	JB8	0.747			
	JB9	0.751			
	JB10	0.719			
	JB11	0.735			
	JB12	0.754			
	JB13	0.727			
	JB14	0.747			
	JB15	0.786			

OC, organizational cynicism; WA, work alienation; LMX, leader-member exchange; JB, job burnout.

**Table 4:** The HTMT ratios.

Variables	Organizational cynicism	Work alienation	LMX	JB
Organizational cynicism				
Work alienation	0.738			
LMX	0.461	0.381		
JB	0.677	0.727	0.330	

**Table 5:** The Fornell-Larcker criterion.

Variables	Organizational cynicism	Work alienation	LMX	JB
Organizational cynicism	**0.757**			
Work alienation	0.677	**0.751**		
LMX	−0.430	−0.348	**0.758**	
JB	0.640	0.667	−0.313	**0.741**

The diagonal entries of the table display the square roots of AVE values, formatted in bold.

To more clearly address the potential issue of conceptual overlap among the core variables, this study also constructed a series of alternative measurement models for comparison: a three-factor model (organizational cynicism, work alienation, and the cynicism dimension of JB); a two-factor model (combining organizational cynicism and the cynicism dimension of JB into a single factor); and a one-factor model (collapsing organizational cynicism, work alienation, and the cynicism dimension of JB into a single factor). The results showed that the three-factor model demonstrated a significantly better fit (*χ*^2^/d*f* = 1.828, SRMR = 0.027, CFI = 0.974, TLI = 0.971, RMSEA = 0.035) than all other alternative models (two-factor: *χ*^2^/d*f* = 3.482, SRMR = 0.052, CFI = 0.922, TLI = 0.915, RMSEA = 0.061; one-factor: *χ*^2^/d*f* = 5.753, SRMR = 0.066, CFI = 0.849, TLI = 0.836, RMSEA = 0.085). This finding indicates that, although organizational cynicism, work alienation, and JB are theoretically interrelated, employees are able to clearly distinguish among these three distinct experiences, thereby supporting the construct validity of the proposed model.

### 3.3 Common method bias

All data used in this research came from participants’ self-reports, raising the possibility of CMB. To address this, a Harman one-factor test was first conducted for preliminary analysis. Exploratory factor analysis revealed that four factors in total possessed eigenvalues above 1. Among these, the first factor accounted for 38.633% of the variance, which remained below the 40% threshold (Podsakoff et al., 2003), preliminarily indicating that CMB was not a serious concern. To enhance the robustness of this conclusion, a more sensitive latent variable control method was further used for testing (Podsakoff et al., 2012). First, the study performed CFA on the measurement model to ensure that latent variables were measured validly, and this model was used as the baseline. On this basis, the baseline model added a common method factor, and a constraint was imposed to ensure that the factor loadings of this factor were equal across all items, thereby constructing the control model.

Due to the nature of ordered categorical data, robust estimation methods are usually used, such as weighted least squares mean and variance adjustment or maximum likelihood estimation with robust standard errors (Brauer et al., 2023; Li, 2016). Because AMOS software, which defaulted to maximum likelihood (ML) estimation, served as the analytical tool for this study, the preliminary skewness and kurtosis tests (see Table 2) indicated approximate normality in the data, satisfying the preconditions for using this method. In these circumstances, even when data were ordinal categorical, employing ML estimation generally produced fairly robust parameter estimates (Kline, 2023). Table 6 reveals satisfactory overall goodness-of-fit for the two models examined (Hu and Bentler, 1998). Decisions regarding model comparison adhered to established practices in invariance testing (Chen, 2007; Cheung and Rensvold, 2002), with none of the empirical gaps approaching those cutoff values. Thus, adding the common method factor did not notably improve model fit, suggesting that CMB did not substantially distort the measurement structure. Nevertheless, it is important to interpret these statistical results with caution. Both the Harman test and the latent factor test are post-hoc statistical remedies, which are generally considered insufficient to definitively rule out the presence of CMB. More critically, no procedural remedies, such as temporal separation, marker variables, or multi-source data collection, were implemented in the current study design. Therefore, while these diagnostic tests did not reveal overt signs of CMB, the possibility that it serves as a potential confounding factor cannot be entirely dismissed. This concern is particularly pertinent to the observed interaction effects, especially the organizational cynicism × LMX interaction, which may be susceptible to inflation due to CMB; future research should therefore employ multi-source or time-lagged designs to more rigorously validate these conditional relationships.

**Table 6:** Model fitting differences.

Index	*χ*^2^/d*f*	SRMR	RMSEA	IFI	CFI	GFI	TLI	NFI	RFI
Reference value	<3	<0.06	<0.06	>0.9	>0.9	>0.9	>0.9	>0.9	>0.9
Baseline model	1.601	0.036	0.030	0.967	0.967	0.911	0.965	0.918	0.912
Control model	1.603	0.036	0.030	0.967	0.967	0.911	0.965	0.918	0.912
Model fit difference	0.002	<0.001	<0.001	<0.001	<0.001	<0.001	<0.001	<0.001	<0.001
Criterion		Δ < 0.025	Δ < 0.01	Δ < 0.01	Δ < 0.01	Δ < 0.01	Δ < 0.02	Δ < 0.01	Δ < 0.01

### 3.4 Collinearity test

This study diagnosed and evaluated the issue of multi-collinearity among the independent variables using the variance inflation factor (VIF). The results in Table 7 showed that the VIF values for all predictor variables were below 5 and the tolerance values were above 0.2, indicating that multi-collinearity was not a concern (Hair et al., 2019).

**Table 7:** The results of collinearity test.

Variables	VIF	Tolerance
Organizational cynicism	1.994	0.502
Work alienation	1.853	0.540
LMX	1.223	0.818

### 3.5 Analysis of differences between groups

To examine differences in JB across different demographic and occupational background characteristics, this study conducted independent samples t-tests and one-way analysis of variance. The results (Table 8) showed that JB differed across groups defined by gender, age, educational background, years of working, and weekly working hours, while no significant differences were found for marital status, department, or position level.

**Table 8:** Test results.

Outcome variable	Covariate		*M* ± SD	*t*/*F*	*p*
JB	Gender	Male (*n* = 330)	2.896 ± 0.796	4.283	<0.001
		Female (*n* = 328)	3.159 ± 0.777		
	Age	25 years and below (*n* = 89)	3.149 ± 0.667	16.116	<0.001
		26–35 years (*n* = 211)	3.280 ± 0.714		
		36–45 years (*n* = 210)	2.964 ± 0.758		
		46–55 years (*n* = 116)	2.775 ± 0.907		
		56 years and above (*n* = 32)	2.354 ± 0.788		
	Education background	High school/Vocational school and below (*n* = 67)	2.741 ± 0.734	5.293	0.001
		Associate degree (*n* = 129)	2.909 ± 0.753		
		Bachelor’s degree (*n* = 300)	3.107 ± 0.797		
		Master’s degree and above (*n* = 162)	3.093 ± 0.825		
	Marital status	Unmarried (*n* = 113)	3.047 ± 0.838	1.001	0.406
		Married without children (*n* = 173)	3.087 ± 0.838		
		Married with children (*n* = 328)	3.012 ± 0.768		
		Divorced (*n* = 36)	2.806 ± 0.801		
		Widowed (*n* = 8)	3.096 ± 0.796		
	Years of working	Less than 1 year (*n* = 68)	3.313 ± 0.660	13.893	<0.001
		1–3 years (*n* = 124)	3.255 ± 0.655		
		4–6 years (*n* = 118)	3.211 ± 0.786		
		7–9 years (*n* = 149)	2.940 ± 0.770		
		10 years and above (*n* = 199)	2.746 ± 0.849		
	Department	Production (*n* = 164)	3.016 ± 0.796	0.612	0.721
		Marketing (*n* = 154)	3.007 ± 0.838		
		Finance (*n* = 123)	3.127 ± 0.771		
		Research and development (*n* = 60)	2.913 ± 0.824		
		Human resources (*n* = 61)	3.025 ± 0.868		
		Administration (*n* = 46)	2.973 ± 0.698		
		Others (*n* = 50)	3.073 ± 0.712		
	Position level	Ordinary employee (*n* = 337)	2.978 ± 0.776	1.941	0.122
		Front-line manager (*n* = 218)	3.111 ± 0.819		
		Middle manager (*n* = 82)	3.070 ± 0.806		
		Senior manager (*n* = 21)	2.791 ± 0.815		
	Weekly working hours (including overtime)	40 h and below (*n* = 174)	2.569 ± 0.776	35.931	<0.001
		41–50 h (*n* = 216)	3.065 ± 0.775		
		51–60 h (*n* = 185)	3.225 ± 0.696		
		Above 60 h (*n* = 83)	3.452 ± 0.662		

Absolute values of *t* is reported.

Specifically, female employees reported significantly higher JB than males (*t*(656) = 4.283, *p* < 0.001, Cohen’s *d* = 0.334), indicating a small effect. For variables with more than two groups, Bonferroni-corrected post-hoc comparisons revealed that: Age (*F*(4,653) = 16.116, *p* < 0.001, *η*^2^ = 0.090): showed a moderate effect, with employees aged 26–35 years scored highest, significantly higher than all groups aged 36 years and above; Education background (*F*(3,654) = 5.293, *p* = 0.001, *η*^2^ = 0.024): showed a small-to-moderate effect, with bachelor’s and master’s degree holders scored higher than those with high school education or below; Years of working (*F*(4,653) = 13.893, *p* < 0.001, *η*^2^ = 0.078): showed a moderate effect, with employees with fewer years of working (especially <7 years) reported higher JB than those with 10+ years; Weekly working hours (*F*(3,654) = 35.931, *p* < 0.001, *η*^2^ = 0.141): demonstrated a large effect and a clear dose–response pattern emerged, with each higher hour category showing progressively higher JB. No significant group differences were found for marital status, department, or position level (all *p* > 0.05).

### 3.6 Analysis of mediating effects

Following the analytical procedures recommended by Keith (2019), this study employed hierarchical regression analysis to examine the hypothesized relationships. The coding and entry strategies for all variables were specified as follows: Gender was dummy-coded with male = 1 and female = 2. Age, educational background, years of working, and weekly working hours were treated as ordinal covariates, coded from low to high based on their original ordered categories (1 = lowest category, with higher scores indicating older age, higher education, longer tenure, and longer working hours, respectively). To maintain parsimonious statistical adjustment and examine the average linear trend across ordered categories, these ordinal variables were entered as approximately continuous covariates. We acknowledge that this approach assumes equal spacing between adjacent ordinal categories, a convention commonly adopted when ordinal categories reflect a clear progressive sequence (Norman, 2010). More importantly, we conducted a robustness check by re-running all regressions with all four control variables entered as sets of dummy codes. The results for all regression coefficients remained consistent in direction and statistical significance, with only minor fluctuations in magnitude, confirming that our substantive conclusions are not contingent upon the equal-interval coding assumption. The complete results are reported in Appendix. Accordingly, the reported coefficients for these ordinal covariates should be interpreted as average changes associated with moving to a higher category, rather than as precise unit effects. To test the mediating role of work alienation, a two-block hierarchical regression was conducted. In Block 1, all control variables along with organizational cynicism were forcedly entered to examine the total effect of organizational cynicism on JB. In Block 2, work alienation was additionally entered on top of Block 1 to examine its independent effect on JB after controlling for organizational cynicism, and to observe the attenuation of the regression coefficient of organizational cynicism. The results are presented in Table 9. In the final model (included all predictors), the effects of each control variable on JB were as follows: gender, education background, years of working, and weekly working hours all demonstrated significant relationships with JB, whereas age did not show a statistically significant association. Among these control variables, weekly working hours exhibited the strongest relative association with JB, suggesting that workload may play a non-negligible role in shaping employees’ JB. Organizational cynicism was significantly positively associated with work alienation and JB. When both work alienation and organizational cynicism were included in the regression equation, work alienation was significantly positively correlated with JB. At this point, the regression coefficient for organizational cynicism decreases from 0.537 to 0.307 (still significant), indicating that work alienation partially mediates the relationship between the two. As shown in Table 10, the 95% confidence interval for the indirect effect did not contain zero, further supporting the presence of the mediating effect. H1, H2, H3, and H4 were all supported.

**Table 9:** The results of regression analysis.

Dependent variable	Independent variable	*β*	*B*	SE	*t*	*p*	95% CI		*R* ^2^	*F*
							LLCI	ULCI		
Work alienation	Organizational cynicism	0.618	0.618	0.031	20.045	<0.001	0.557	0.678	0.480	100.074***
JB	Organizational cynicism	0.537	0.537	0.031	17.530	<0.001	0.477	0.597	0.487	102.834***
	Gender	0.065	0.131	0.053	2.471	0.014	0.027	0.234	0.558	117.416***
	Age	−0.025	−0.023	0.026	−0.883	0.377	−0.075	0.028		
	Education background	0.056	0.061	0.029	2.115	0.035	0.004	0.118		
	Years of working	−0.097	−0.071	0.020	−3.538	<0.001	−0.111	−0.032		
	Weekly working hours	0.177	0.179	0.028	6.512	<0.001	0.125	0.233		
	Organizational cynicism	0.307	0.307	0.036	8.501	<0.001	0.236	0.378		
	Work alienation	0.372	0.372	0.036	10.280	<0.001	0.301	0.442		

*B*, unstandardized coefficient; SE, standard error; CI, confidence interval; *β*, standardized coefficient. SE, *t*, and CI are reported for *B* only; *β* is provided for effect-size comparison without inferential statistics. Organizational cynicism, work alienation, and JB were *Z*-score standardized, making *B* and *β* numerically equivalent for these independent variables.

**Table 10:** Mediation effect analysis.

Effect	Path	*β*	Percentage (%)	95% CI		Mediation type
				LLCI	ULCI	
Total effect	Organizational cynicism → JB	0.537	100%	0.477	0.597	
Direct effect	Organizational cynicism → JB	0.307	57.17%	0.236	0.378	
Indirect effect	Organizational cynicism → work alienation → JB	0.230	42.83%	0.179	0.285	Partial Mediation

### 3.7 Analysis of moderating effects

To probe the significant interaction, we estimated conditional effects (simple slopes) at two representative values of the continuous moderator LMX: at one SD beneath the average (*M* − 1SD) and at one SD above the average (*M* + 1SD). Importantly, LMX was retained as a continuous variable throughout all analyses; the ‘low’ and ‘high’ designations are used solely for the purpose of simple slope estimation and data visualization. Table 11 presents the results of the moderating effect test after controlling for variables with inter-group differences (gender, age, educational background, years of working, and weekly working hours, same as Table 9). Both the interaction terms of organizational cynicism and LMX were significantly negatively correlated with work alienation and JB. According to this finding, LMX significantly and negatively moderated two relationships: that between organizational cynicism and work alienation, and that between organizational cynicism and JB. Bootstrap analyses (5,000 resamples) revealed that the conditional indirect effects of organizational cynicism on JB via work alienation were significant across all LMX levels, with effect sizes decreasing as LMX increased. The index of moderated mediation was negative with a confidence interval excluding zero, confirming that LMX significantly and negatively moderates the mediation process (see Table 12 for detailed results). Taken together, these findings provide complete statistical support for the moderated mediation model. H5 and H6 were supported. See Figure 2 for the path analysis schematic of the model.

**Table 11:** The moderating role of leader-member exchange.

Dependent variable	Independent variable	*β*	SE	*T*	95% CI		*R* ^2^
					LLCI	ULCI	
Work alienation	Organizational cynicism	0.552	0.034	16.075***	0.484	0.619	0.497
	LMX	−0.112	0.032	−3.547***	−0.175	−0.050	
	Organizational cynicism × LMX	−0.137	0.037	−3.663***	−0.210	−0.063	
	Gender	0.076	0.057	1.349	−0.035	0.188	
	Age	−0.055	0.028	−1.936	−0.111	0.001	
	Education background	0.009	0.031	0.287	−0.052	0.070	
	Years of working	−0.073	0.022	−3.375***	−0.115	−0.031	
	Weekly working hours	0.096	0.029	3.255***	0.038	0.153	
JB	Organizational cynicism	0.278	0.037	7.428***	0.205	0.352	0.572
	LMX	−0.070	0.030	−2.385*	−0.128	−0.012	
	Organizational cynicism × LMX	−0.146	0.035	−4.179***	−0.214	−0.077	
	Work alienation	0.343	0.036	9.451***	0.271	0.414	
	Gender	0.140	0.052	2.669**	0.037	0.243	
	Age	−0.041	0.026	−1.575	−0.093	0.010	
	Education background	0.057	0.029	1.996*	0.001	0.113	
	Years of working	−0.078	0.020	−3.888***	−0.118	−0.039	
	Weekly working hours	0.168	0.027	6.147***	0.115	0.222	

Path significance: ***p < 0.001; **p < 0.01; *p < 0.05.

**Table 12:** Conditional indirect effects and index of moderated mediation.

LMX level	Conditional indirect effect	SE	95% CI		Index of moderated mediation	SE	95% CI	
			LLCI	ULCI			LLCI	ULCI
Low (*M* − 1SD)	0.236	0.033	0.175	0.302	−0.047	0.017	−0.081	−0.014
Mean	0.189	0.026	0.141	0.242				
High (*M* + 1SD)	0.142	0.029	0.089	0.205				

**Figure 2:**
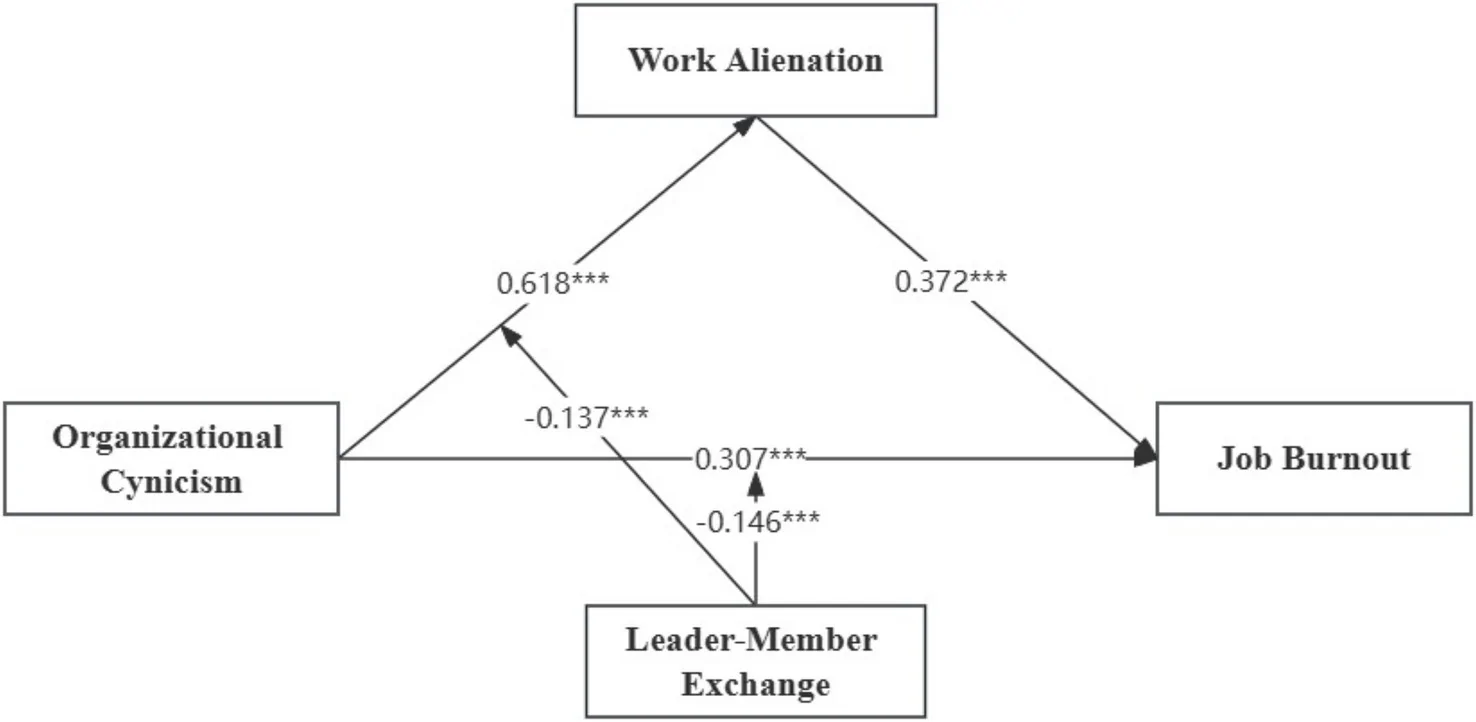
Path analysis diagram.

The simple slope analysis graphs (see Figures 3 and 4) provided visual support for the buffering effect, which we further quantified to aid practical interpretation. At high levels of LMX (+1 SD), the simple slope of organizational cynicism on work alienation was *β* = 0.415, whereas at low levels of LMX (−1 SD), it was *β* = 0.689, a difference of 0.274 in standardized coefficients. This indicates that the positive association between organizational cynicism and work alienation was substantially weaker in the high-quality LMX group compared to the low-quality LMX group. In other words, for every one-SD increase in LMX, the positive effect of organizational cynicism on work alienation decreases by 0.137 in slope magnitude. Similarly, for JB, the simple slope of organizational cynicism on JB decreased from *β* = 0.424 (low LMX) to *β* = 0.132 (high LMX), yielding a difference of 0.292 in standardized coefficients. That is, for every one-SD increase in LMX, the positive effect of organizational cynicism on JB decreases by 0.146 in slope magnitude. Following the benchmarks of Cohen (1988), these effect-size differences (0.27–0.29 SD) fall within the small-to-medium range, suggesting that the buffering role of LMX carries practically meaningful implications for workplace interventions.

**Figure 3:**
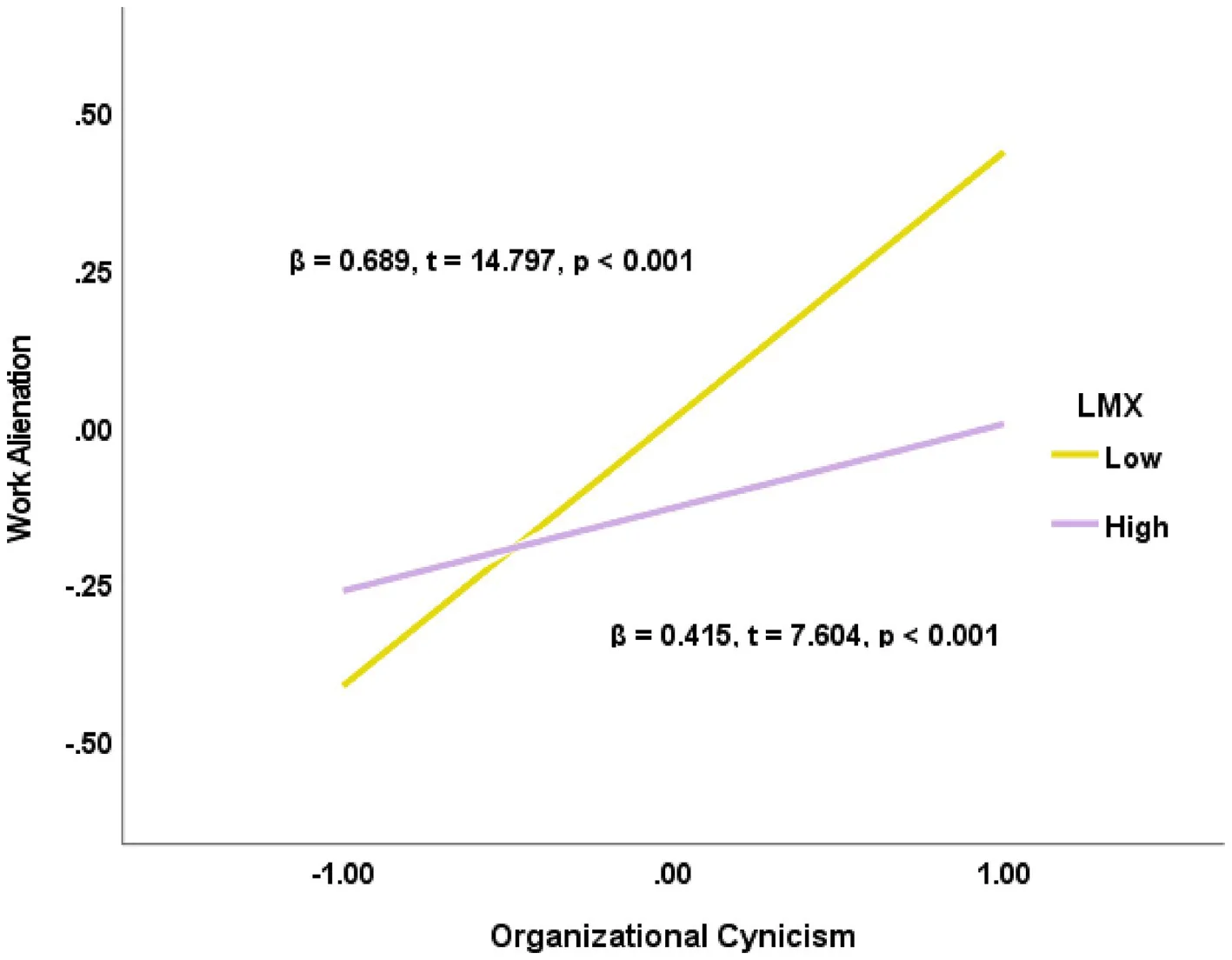
The moderating role of LMX on the relationship between organizational cynicism and work alienation.

**Figure 4:**
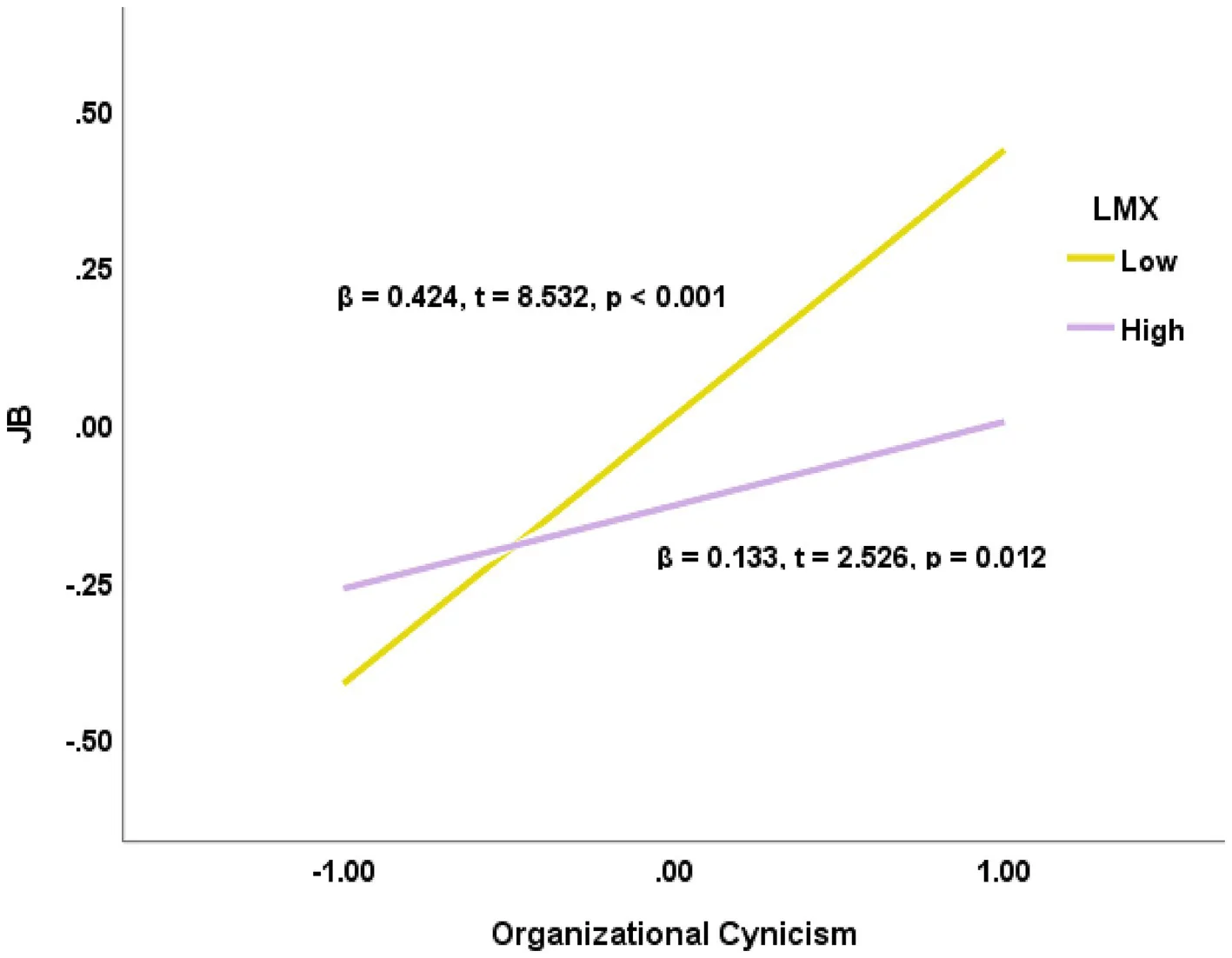
The moderating role of LMX on the relationship between organizational cynicism and JB.

Furthermore, this research applied the Johnson-Neyman approach to better delineate the moderating effect’s boundary conditions. The results showed that LMX had a critical threshold effect on the strength changes of the above-mentioned relationship (see Figures 5 and 6). Specifically, for JB, the positive association between organizational cynicism and JB remained significant when LMX was below the threshold value (*Z* < 1.151), but became non-significant when LMX exceeded this critical value (*Z* > 1.151). This indicates that high-quality LMX functions as a buffer in the relationship between organizational cynicism and JB. Importantly, the Johnson-Neyman boundary identified here denotes the point at which the conditional association crossed the threshold of statistical significance (*p* = 0.05), rather than the point at which the buffering effect or conditional slope became zero (Bauer and Curran, 2005; Preacher et al., 2006). Above this threshold, although the positive association between cynicism and burnout was no longer statistically distinguishable from zero, the estimated simple slope remained a nonzero value; the non-significance reflects sampling variability rather than a nil effect. For work alienation, however, a different pattern emerged. Although the positive relationship between organizational cynicism and work alienation gradually weakened as LMX increased, it remained statistically significant across all observed levels of LMX, including at the highest level (*Z* = 1.862). In other words, the Johnson-Neyman procedure did not yield a crossing point within the data range for this outcome, suggesting that the buffering effect of LMX on work alienation, while meaningful in magnitude, is insufficient to completely eliminate the positive association within the sampled range. Together, these region-of-significance findings suggest that high-quality LMX shows a stronger mitigating effect on the risk of JB associated with organizational cynicism, whereas its protective function against work alienation is comparatively limited, with the positive association persisting even at higher levels of relational quality. This nuanced interpretation, supported by both graphical and numerical outputs of the Johnson-Neyman technique, enhances the understanding of when and for which outcomes LMX exerts its moderating role.

**Figure 5:**
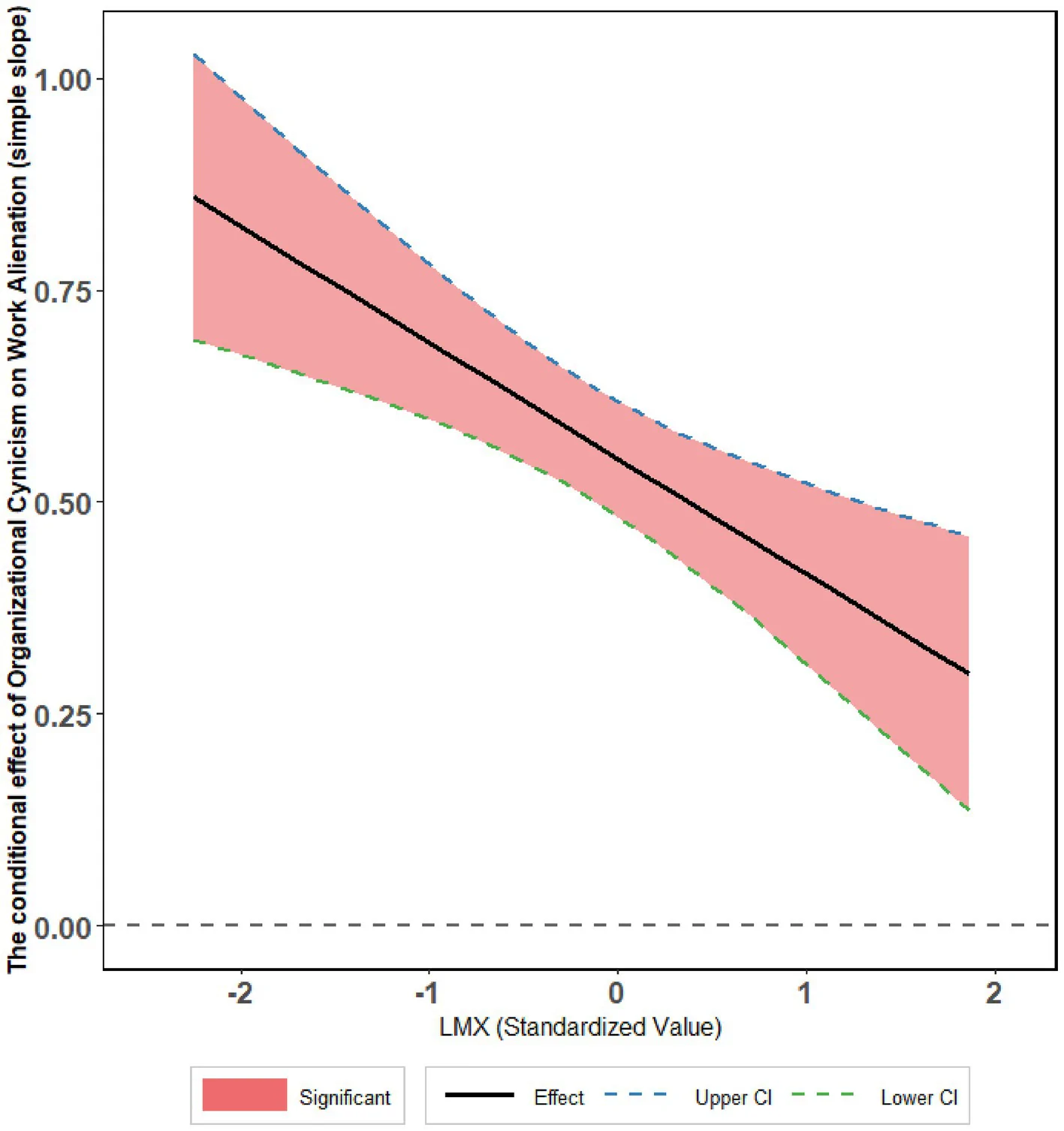
Johnson-Neyman analysis of the moderating effect of LMX on the relationship between organizational cynicism and work alienation.

**Figure 6:**
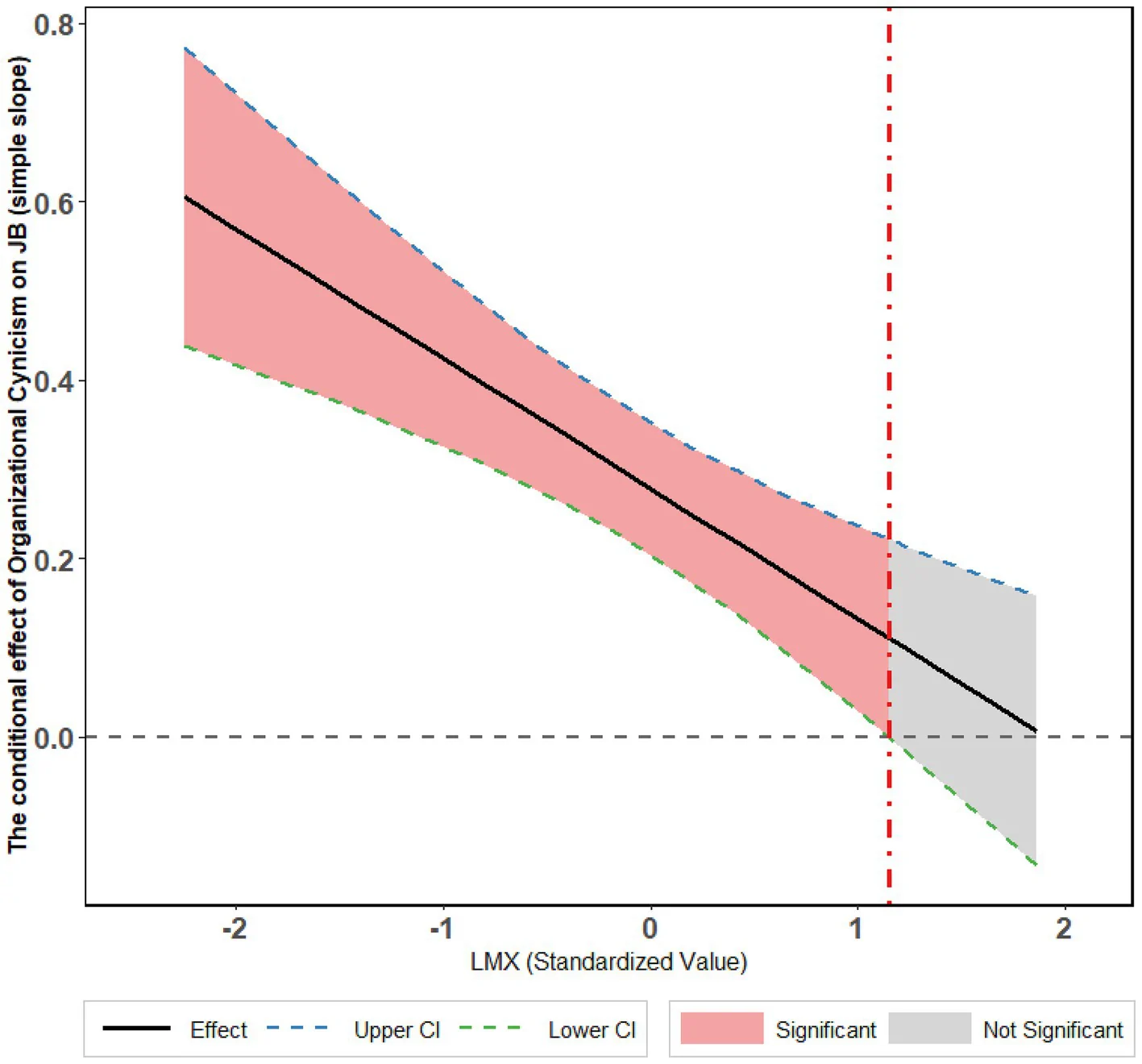
Johnson-Neyman analysis of the moderating effect of LMX on the relationship between organizational cynicism and JB.

## 4 Discussion

Organizational cynicism was significantly positively associated with employees’ JB, meaning that the more skepticism and disillusionment employees held toward organizational values, management practices, or change motives, the higher their level of JB. This finding corroborates the results of Kim et al. (2025). From the perspective of COR theory (Hobfoll, 1989), organizational cynicism is not merely an attitudinal state but essentially reflects employees’ ongoing assessment of the depletion of resources related to organizational fairness and integrity. When employees repeatedly encounter situations where the organization’s words and actions do not align or where its motives are questionable, their trust in the organization continues to erode (Okçu et al., 2023). Our findings offer a tentative COR-based account of this association: to prevent further resource loss, employees tend to adopt indifferent coping strategies, manifested as depersonalization and emotional exhaustion, which are the core symptoms of JB (Salama et al., 2025). During this process, the fit between people and the organization could establish a work setting that cultivates organizational trust, and employees usually experience a lower level of burnout symptoms (Flatau-Harrison et al., 2025). Conversely, when cynicism becomes embedded in an organization’s culture as a form of routine, institutionalized disappointment, employees perceive not a negotiable opportunity for improvement, but rather an unchangeable atmosphere of hypocrisy. In such a context, burnout rates are significantly higher (Karaman et al., 2025). This contrast suggests that the cynicism-burnout link is plausibly interpreted as a consequence of sustained psychological contract breach and prolonged resource imbalance, particularly when the organizational trust system remains fragile over the long term.

Organizational cynicism showed a significant positive association with work alienation, and work alienation was significantly positively associated with employees’ JB. Mediation analysis further revealed that work alienation played a partial mediating role between the two. This result is consistent with the research of Demir and Inan (2025), revealing the psychological process of employees from organizational cynicism to the deepening of JB. In the context of prolonged organizational cynicism, employees’ trust in organizational fairness and integrity is steadily eroded; this psychological wear and tear serves as the underlying condition for the deepening of their sense of work alienation (Singh and Randhawa, 2022). Alienation itself cuts off the sense of meaning and intrinsic motivation derived from work, leaving individuals with low levels of disposable resources at the emotional and cognitive levels, thereby manifesting significant burnout symptoms (Galal et al., 2026). Notably, while our data are consistent with this process, we must acknowledge that our mediation analysis is based on cross-sectional data and thus cannot establish the temporal precedence required for causal mediation. We further speculate that the Chinese organizational context may offer a particularly instructive setting for extending COR theory (Hobfoll, 2001), although this speculation awaits direct empirical testing. Against the backdrop of collectivist culture that values harmony and avoids conflict, employees tend to internalize rather than openly express dissatisfaction. This implicit depletion occurs. Psychological alienation is at a high level at the same time. Moreover, because work meaning is deeply tied to family responsibility and collective belonging in the Chinese context, the sense of meaninglessness under alienation may manifest as a collapse of overall life value, a more profound loss than typically observed in Western contexts, where individualism may allow for more psychological detachment. However, because we did not directly measure cultural values (e.g., collectivism–individualism at the individual level) nor include a cross-cultural comparison sample, these cultural interpretations remain speculative. Therefore, we suggest that, if replicated in future research using culturally diverse samples, interventions targeting JB in Chinese organizational context might benefit from not only improving organizational fairness and communication, but also addressing cultural–psychological factors to help employees rebuild the meaningful connection between work and self.

This study tested whether LMX moderates the relationship of organizational cynicism with work alienation and also with JB, and all hypotheses were supported. Further analysis indicated that the interaction between LMX and organizational cynicism on work alienation and JB was significant: when the quality of LMX was high, even if employees held higher organizational cynicism, their sense of job alienation and the degree of JB were relatively low. This pattern can be tentatively explained from the perspective of COR theory (Hobfoll, 2001): high-quality LMX provides employees with additional relational resources, such as emotional support and information sharing, thereby creating a psychological buffer between employees and the organization. Under conditions of resource threat from organizational cynicism, these supplementary resources appear to be associated with reduced core resource depletion, and correspond to reduced conversion of cynical emotions into work alienation and deviant workplace behaviors. We speculate that, in the Chinese cultural context, this moderating effect might be more prominent, a hypothesis that remains to be directly tested. Under the Confucian norms that value interpersonal harmony, hierarchical order, and superior-subordinate trust, employees who have established good interactive relationships with their leaders might be more likely to interpret organizational injustices or negative phenomena as temporary or localized issues rather than completely denying the connection between themselves and the organization. Conversely, employees experiencing poorer quality of LMX lack such relational resources, making it more probable to solidify cynicism into a detached attitude toward the work itself (Jiang et al., 2019). It is worth noting that the above findings further refine the boundary conditions of LMX’s role. Although LMX significantly protects against the transformation of organizational cynicism into work alienation and JB, it does not completely overturn the established relationship between organizational cynicism and work alienation. This finding suggests that LMX primarily plays a buffering rather than a cognitive restructuring role: it is better at establishing a barrier between negative emotions and negative behaviors rather than directly transforming employees’ fundamental cognitive framework regarding organizational cynicism (Li et al., 2021). From an alternative angle, however, it is also possible that high-quality LMX does not simply buffer resource loss but may, in a more complex twist, intensify emotional labor demands. Employees enjoying close leader relationships might experience a heightened sense of obligation to sustain positive affect and suppress cynical expressions, a dynamic that, over time, could paradoxically erode rather than preserve emotional resources (Grandey, 2000; Humphrey et al., 2015). While our buffering interpretation is consistent with the data, this competing explanation suggests that the protective function of LMX may have upper limits or even contingent costs, a boundary condition that future research should explore. To empirically disentangle these two countervailing pathways, future studies could directly measure employees’ use of surface acting, the suppression of genuine feelings and faking of required emotions, as a key indicator of emotional labor intensification. If surface acting mediates or moderates the relationship between LMX and JB, it would provide evidence for the intensification route, whereas its absence would support the buffering account. Additionally, incorporating other emotional labor strategies (e.g., deep acting) as parallel mediators within a multi-wave, multi-source design could further compare the relative magnitude of these competing effects, thereby revealing the net effect of LMX on JB.

Regarding demographic and job-related differences, employees of different genders, ages, educational background, years of working, and weekly working hours exhibit significant between-group differences in the levels of JB. Specifically, female employees score higher in JB, which we tentatively attribute to insufficient resource compensation under the dual-role pressure of family and work (Fang et al., 2024). Younger employees, who are still in the early stages of their careers and have not yet developed a strong sense of organizational belonging, exhibit relatively high levels of JB. In contrast, older employees generally exhibit lower levels of JB (Fetter and Dixon, 2025; Yu et al., 2024), likely due to greater psychological resilience and more adaptive work values accumulated over their careers. Unlike findings in Western contexts (Cvjetkovic et al., 2026; Pihl-Thingvad et al., 2025), highly educated Chinese employees possess richer cognitive resources yet hold higher expectations for work meaning and autonomy. Once organizational support falters, the gap between ideals and reality can escalate into deep-seated burnout, suggesting that cognitive resources without corresponding organizational support can paradoxically intensify resource loss spirals. Regarding years of working, employees in the early career stage show higher levels of burnout, reflecting the inherent conflict between high expectations and high intensity pressure at the initial stage of employment, whereas burnout levels gradually decrease with increasing work tenure (Obeidat et al., 2026). In addition, among employees at the surveyed companies, nearly 75% reported working overtime, and this group exhibited significantly higher levels of burnout than those who did not work overtime (Aziz and Ong, 2024; Nowacka et al., 2025). This finding suggests that overtime work accelerates resource depletion by curtailing sufficient recovery time, thereby exacerbating burnout. Viewed through the lens of COR theory, these demographic differences reflect systematic variation in resource reservoirs: younger employees lack accumulated resource caravans that buffer burnout, while overtime work accelerates resource depletion by curtailing recovery, and female employees face dual-domain resource drain from family and work demands. Viewed alongside our moderated mediation model, these patterns suggest that employees with fewer baseline resources may be particularly sensitive to both the erosive effects of organizational cynicism and the buffering benefits of high-quality LMX, implying that demographic factors could serve as important contextual boundary conditions for the effectiveness of workplace interventions.

## 5 Implications and limitations

### 5.1 Theoretical implications

Grounded in COR theory, this study explores the intrinsic association between organizational cynicism and employees’ JB, providing a testable theoretical framework for future research. Firstly, the research reveals how organizational cynicism, an external negative perception, is systematically associated with the internal psychological process of work alienation and the negative emotional outcome of JB. Second, the study identified the dual moderating conditions of LMX and found that high-quality LMX, as a critical external resource, serves as a buffer during both the early stage (the formation of alienation) and the late stage (the onset of burnout) of negative cognitive transformation, thereby providing a theoretical basis for understanding when and how to mitigate the negative effects of negative cognition. Within this theoretical framework, the study constructs an explanatory model integrating external cognitive factors, internal psychological mechanisms, and situational moderating conditions, deepening the theoretical understanding of the dynamic mechanisms underlying employees’ JB formation. Furthermore, the research has revealed the differentiated distribution characteristics of demographic and work background variables in JB. This finding suggests that in future studies, when constructing theoretical models of JB, demographic characteristics should be considered as important control or stratification variables. In summary, this study systematically elucidates the mechanisms underlying employees’ JB and the associated group differences from the perspective of the integration of external negative cognitions and internal psychological resources within a contextual framework, thereby providing a theoretical foundation for advancing research in occupational health psychology within the field of organizational management.

### 5.2 Practical implications

This study revealed the underlying mechanism linking organizational cynicism and employees’ JB, providing the following practical implications for organizational management. First, prioritize early identification and intervention regarding cynical sentiments. Managers should adopt concrete measures such as anonymous surveys (e.g., quarterly short-form questionnaires), semi-structured focus groups, or one-on-one conversations to detect employees’ dissatisfaction and skepticism about organizational policies, culture, or management practices at an early stage. For employees with high cynicism tendency, managers should schedule dedicated feedback sessions within 2 weeks of identification, using active listening and problem-solving dialogs to prevent the escalation of work alienation and emotional exhaustion. Second, block the key transmission path of work alienation. For example, hold monthly “achievement showcase” meetings where teams present successful project outcomes, as frequent positive feedback and recognition events have been shown to reinforce self-efficacy and perceived impact (Grant and Sonnentag, 2010); organize quarterly experience-sharing circles where employees discuss how their roles contribute to organizational goals; and facilitate cross-departmental collaboration days to enhance role clarity and perceived task significance. Third, enhance the quality of LMX to exert a buffering effect. Specific actions include: designing a quarterly LMX-focused leadership training module that emphasizes empathetic communication, active feedback, and individualized consideration, a frequency supported by evidence that spaced, multi-session interventions yield superior behavioral transfer compared to one-time events (Lacerenza et al., 2017); encouraging managers to hold weekly 15-min one-on-one check-ins with each subordinate to tailor support based on personal needs; and integrating the quality of LMX, measured by subordinate ratings and behavioral indicators, into managers’ annual performance appraisals and 360-degree feedback systems, with concrete improvement plans for low-scoring leaders. Fourth, implement differentiated, tiered intervention strategies for high-risk groups. For young or junior employees, strengthen onboarding buddy systems, structured mentoring programs (with monthly goal reviews), and individualized career development roadmaps. For highly educated employees, offer elective professional autonomy initiatives, such as self-directed project selection and access to advanced skill-building workshops. For groups with excessively long weekly working hours, enforce mandatory overtime caps, promote flexible scheduling, and pilot remote-work options with clear output-based performance criteria. For female employees, proactively address work–family balance by providing on-site childcare subsidies, flexible start/end times, and periodic workload adjustments during peak family-care periods. Fifth, shift the management of JB from passive response to proactive prevention. Establish a routine organizational health surveillance system, including bi-annual well-being audits using validated burnout scales, and create a real-time anonymous reporting channel for stress-related concerns. For groups identified as high-risk through these audits, offer on-site or virtual counseling sessions (at least twice per month), structured stress-management workshops (e.g., mindfulness-based resilience training) (Liu et al., 2023), and managerial debriefings to adjust workload distribution or task design accordingly. These prevention-oriented policies should be reviewed and updated semiannually based on outcome data.

### 5.3 Limitations and future directions

This study preliminarily examined the intrinsic association between organizational cynicism and employees’ JB, but several limitations should be noted when generalizing the findings. First, the research adopted a cross-sectional design. Although the model proposes explicit path hypotheses based on COR theory and tests the theoretical relationships among variables through path analysis, the mediated relationships we observed are correlational in nature and cannot establish temporal precedence. This means that the indirect effects we report may be biased by unmeasured time-varying confounds, and any causal interpretation would be unwarranted. Future research could employ longitudinal designs, such as follow-up surveys or intervention experiments, to more effectively reveal causal relationships and dynamic evolution processes among variables. Secondly, all variables in this study were collected through self-report questionnaires at a single time point, and no procedural remedies were employed. Although this study employed Harman’s one-factor test and latent common method factor analysis for statistical diagnostics, these post-hoc statistical controls, while informative, cannot fully substitute for procedural precautions in eliminating the influence of CMB. Consequently, we cannot rule out the possibility that CMB has inflated the observed associations, particularly the interaction and mediation effects, due to shared response tendencies. Future research could integrate data from multiple sources, such as peer evaluations, supervisor ratings, and attendance records, to enhance the objectivity of the measurements and the interpretability of the findings. Third, convenience sampling was used in this study, possibly underrepresenting hard-to-reach groups, such as employees of small and micro enterprises in remote areas or older employees who rarely use digital devices. This sampling constraint means that our observed effect sizes may reflect the characteristics of a relatively accessible, digitally literate, and urban-leaning subpopulation, which could differ systematically from the broader workforce in terms of work values, organizational commitment, and cynicism expression. Consequently, the magnitude and even the direction of the relationships we report may not hold for underrepresented groups. Therefore, caution should be exercised when extending the research conclusions to these groups. Future research should attempt to adopt more systematic sampling strategies to enhance the representativeness of the sample and the external validity of the findings. Fourth, the sample of this study was entirely drawn from employed workers in the Chinese organizational context, where high power distance, collectivism, and guanxi-based relational norms prevail. This cultural specificity limits the generalizability of our findings. The buffering effect of LMX we observed may be particularly salient in such a high-power-distance setting, where supervisor-subordinate relationships are heavily affect-laden. If this pattern extends to low-power-distance or individualistic cultures (e.g., Scandinavian or Anglo-Saxon settings), where employment relationships are more transactional, then the buffering effect of LMX is likely to be weaker, because subordinates in such contexts may not rely as heavily on the socioemotional resources from supervisors to cope with workplace stressors. Therefore, our reported effect sizes, especially for the LMX interaction, should be treated as context-dependent rather than universal, and cross-cultural replications are needed to determine the boundaries of our findings. In addition, age, educational background, years of working, and weekly working hours were represented using ordered-category codes and treated as approximately continuous covariates. Although this approach provided parsimonious statistical adjustment, it assumed approximately linear trends across adjacent categories and may not fully capture nonlinear or category-specific differences, Future research could use dummy coding or more detailed continuous measures to examine these demographic and occupational characteristics.

## 6 Conclusion

Grounded in COR theory, this study systematically examined the associations among organizational cynicism, work alienation, LMX, and employees’ JB. The results showed that organizational cynicism was significantly positively associated with JB, with work alienation playing a partial mediating role. LMX played a negative moderating role in both organizational cynicism and work alienation, as well as organizational cynicism and JB. In addition, significant differences in employees’ JB were observed across groups based on gender, age, educational background, years of working, and weekly working hours. These findings not only deepen the understanding of the cognitive and emotional transformation processes in the formation of JB, but also provide practical references for mitigating the risk of employees’ JB and enhancing organizational health and sustainable development capabilities. It should be noted that this study mainly focuses on the cognitive, emotional and individual-level interpersonal relationship elements, without a systematic investigation of broader macro-organizational background variables. Future research should integrate the macro-organizational context with micro-psychological processes, building a more thorough theoretical model across multiple levels.

## Data Availability

The raw data supporting the conclusions of this article will be made available by the authors, without undue reservation.

## Footnotes

1
https://www.sojump.com/

## Appendix

**Table A1:** Robustness check results using dummy coding for ordinal variables.

Dependent variable	Independent variable	*β*	*B*	SE	*t*	*p*	95% CI		*R* ^2^	*F*
							LLCI	ULCI		
Work alienation	Organizational cynicism	0.620	0.620	0.031	19.864	<0.001	0.558	0.681	0.498	39.750***
JB	Organizational cynicism	0.532	0.532	0.031	17.108	<0.001	0.471	0.594	0.500	40.136***
	Gender	0.063	0.127	0.053	2.393	0.017	0.023	0.231	0.567	49.238***
	Age_2	0.063	0.136	0.085	1.588	0.113	−0.032	0.304		
	Age_3	0.001	0.001	0.086	0.016	0.988	−0.168	0.171		
	Age_4	−0.004	−0.012	0.097	−0.121	0.904	−0.203	0.179		
	Age_5	0.007	0.031	0.145	0.216	0.829	−0.254	0.317		
	Education background_2	0.048	0.121	0.101	1.192	0.234	−0.078	0.319		
	Education background_3	0.104	0.209	0.091	2.284	0.023	0.029	0.388		
	Education background_4	0.085	0.197	0.098	2.018	0.044	0.005	0.389		
	Years of working_2	−0.035	−0.091	0.102	−0.893	0.372	−0.290	0.109		
	Years of working_3	−0.004	−0.012	0.103	−0.114	0.910	−0.214	0.190		
	Years of working_4	−0.051	−0.120	0.101	−1.190	0.235	−0.319	0.078		
	Years of working_5	−0.134	−0.292	0.097	−3.017	0.003	−0.481	−0.102		
	Weekly working hours_2	0.108	0.229	0.071	3.252	0.001	0.091	0.368		
	Weekly working hours_3	0.153	0.341	0.074	4.636	<0.001	0.197	0.486		
	Weekly working hours_4	0.192	0.579	0.093	6.206	<0.001	0.396	0.762		
	Organizational cynicism	0.307	0.307	0.037	8.335	<0.001	0.235	0.380		
	Work alienation	0.363	0.363	0.037	9.892	<0.001	0.291	0.435		
